# Framing action to reduce health inequalities: what is argued for through use of the ‘upstream–downstream’ metaphor?

**DOI:** 10.1093/pubmed/fdab157

**Published:** 2021-05-27

**Authors:** Naoimh E McMahon

**Affiliations:** National Institute for Health Research School for Public Health Research (NIHR SPHR), Division of Health Research, Lancaster University, Lancaster, LA1 4YW, UK

**Keywords:** framing, health inequalities, social determinants, upstream

## Abstract

**Background:**

Public health insights struggle to compete with dominant ideas which frame health inequalities as a problem of individual behaviour. There is consequently a need to critically reflect upon and question the effectiveness of different strategies for framing and communicating key insights. Taking the example of the ‘upstream–downstream’ metaphor, this literature review contributes to a necessary first step by asking what exactly is being argued for through its use.

**Methods:**

An iterative search strategy was used to identify peer-reviewed articles which could contribute to the review question. A discourse analysis framework informed data extraction and synthesis of 24 articles. Articles were subsequently categorized into groups which reflected the different uses of the metaphor identified.

**Results:**

All authors used the metaphor to promote a particular causal understanding of health inequalities, leading some to recommend policies and programmes, and others to focus on implementation processes. This seemingly simple metaphor has evolved beyond differentiating ‘upstream’ from ‘downstream’ determinants, to communicate an ambitious politically engaged agenda for change.

**Conclusions:**

The metaphor is not without its critics and in light of the complexity of the arguments encapsulated in its use, work is needed to establish if it can, and does, resonate as intended with wider audiences.

## Introduction

Whether it be policy analysis,^1^ qualitative studies of policy-maker or practitioner perspectives,^2–5^ or evaluations of local action to reduce health inequalities,^3^^,^^6^^,^^7^ research consistently shows how public health insights into the social origins of health and disease struggle to compete with dominant behavioural perspectives, and are consequently limited in their ability to influence thinking and action. These findings pose a challenge to anyone concerned to see a narrowing of health inequalities: to move beyond lamenting the pervasive influence of dominant perspectives and to critically reflect upon and question how different strategies for framing and communicating public health insights work (or indeed fail to work) to influence wider audiences. Taking the example of the ‘upstream–downstream’ metaphor, this article contributes to a necessary first step by establishing what authors are arguing for when they employ this metaphor.

Metaphors are well-established communication devices, which encourage us to understand ‘one kind of thing or experience in terms of another’,^8^ where the latter is often something more familiar or more easily understood. To borrow from Entman’s^9^ definition of framing, metaphors also allow us ‘to select some aspects of a perceived reality and make them more salient in a communicating text, in such a way as to promote a particular problem definition, causal interpretation, moral evaluation and/or treatment recommendation for the item described’. Despite their apparent simplicity and intuitive appeal^10^, however, metaphors risk being interpreted differently by experts within disciplines, and just like key concepts and ideas, can be subject to losing their intended meaning and function as they move from the margins of debate into mainstream use.^11–13^ It is therefore of value to interrogate how metaphors are intended to function, so that we might better understand the extent to which they can, and do, achieve these objectives when deployed amongst wider audiences.

Described by some as the discipline’s ‘defining metaphor’,^14^ the ‘upstream–downstream’ metaphor gained prominence in the form of a story in an influential article by John B. McKinlay^15^:

There I am standing by the shore of a swiftly flowing river and I hear the cry of a drowning man. So I jump into the river, put my arms around him, pull him to shore and apply artificial respiration. Just when he begins to breathe, there is another cry for help. So, I jump into the river, reach him, pull him to shore, apply artificial respiration, and then just as he begins to breathe, another cry for help. So back in the river again, reaching, pulling, applying, breathing, and then another yell. Again and again, without end, goes the sequence. You know, I am so busy jumping in, pulling them to shore, applying artificial respiration that I have *no* time to see who the hell is upstream pushing them all in. [emphasis in original]

While perhaps most often used to differentiate between ‘upstream’ and ‘downstream’ determinants of health, over time the metaphor has been reinterpreted and it is said to have evolved ‘from parable to concept, noun to adjective, and ideal to strategy’,^16^ where it is now not unusual to see phrases such as ‘working upstream’^17^ or ‘moving upstream’.^18^^,^^19^ The purpose of this article is to answer the question: what are authors arguing for when they employ this ‘action-oriented’ use of the metaphor in the health equity literature.

## Methods

The literature review eligibility criteria were designed to identify texts which could best answer the review question. Eligible articles were those that were peer-reviewed and published in English, which focused on health inequalities, and where the ‘upstream–downstream’ metaphor was central to authors’ arguments about the nature of action needed to reduce inequalities. No date restrictions were applied. Initial attempts to identify articles through a highly structured and systematic database search proved impractical, as articles had to be read in full to establish whether they could contribute to the review question, a challenge often experienced in interpretative reviews^20^^,^^21^ (e.g. critical interpretative synthesis). As a result, an iterative approach was adopted using the following information sources: a narrow search of a single database (PubMed); forward and backward citation tracking of articles already known to me, searching of reference lists of potentially relevant and included articles, and searches of author publication lists. Searches were performed in July 2017, and again in June 2020. PubMed was searched using the following search string: ((inequalit*[Title/Abstract] OR inequit*[Title/Abstract] OR equit*[Title/Abstract]) AND upstream[Title/Abstract])). The author (NMcM) was responsible for reading potentially relevant articles in full, applying the eligibility criteria, and selecting articles for inclusion in the review.

Guided by a discourse analysis^22^ framework, NMcM developed and piloted a bespoke data extraction form which was refined over time to ask four key questions of each article: (i) how have authors framed the problem of health inequalities, (ii) how is action to reduce health inequalities framed through use of the metaphor, (iii) what are authors wanting to achieve or concerned to address in using the metaphor in this way and (iv) what wider perspectives or narratives are drawn upon in making the arguments. Relevant text for each question was extracted and summary annotations made using this form. The approach to synthesis involved NMcM threading together the insights from the data extraction forms to produce a narrative account of what authors were arguing for when they employed the ‘upstream–downstream’ metaphor. In light of the interpretative nature of this review, the resulting account should be taken as just one possible reading of a complex body of literature.

Some articles that initially seemed relevant were found during data extraction not to be well placed to contribute to the review. Akin to theory-driven reviews (e.g. realist synthesis^21^), the final 24 articles (or which 14 were retrieved through scoping searches, 8 through PubMed and a further 2 identified when these searches were updated in June 2020) is not an exhaustive list, but represents the richest examples of the how the metaphor is used in academic arguments. An overview of included studies is provided in Table 1. For clarity, the groups are shown as relatively distinct, but there were examples where multiple problem definitions, framings and arguments were present within single articles.

**Table 1:** Overview of included articles and actions argued for through use of ‘upstream–downstream’ metaphor

*Actions argued for through use of the metaphor*		*Author (Year)*	*Title*
‘Upstream’ policies and programmes	Population approach policies (e.g. regulation of industry)	Baelum (2011)^23^	Dentistry and population approaches for preventing dental diseases.
		Capewell & Capewell (2018)^24^	An effectiveness hierarchy of preventive interventions: neglected paradigm or self-evident truth?
		McGill et al. (2015)^25^	Are interventions to promote healthy eating equally effective for all? Systematic review of socioeconomic inequalities in impact.
		Lorenc et al. (2013)^26^	What types of interventions generate inequalities? Evidence from systematic reviews.
	Redistributive policies (e.g. increases in minimum wage)	Dopp & Lantz (2020)^18^	Moving upstream to improve children's mental health through community and policy change.
		Kaplan (2002)^27^	Upstream approaches to reducing socioeconomic inequalities in health.
		SmithBattle (2012)^28^	Moving policies upstream to mitigate the social determinants of early childbearing.
		Whitehead and Popay (2010)^29^	Swimming upstream? Taking action on the social determinants of health inequalities.
	Programmes that account for social norms/power relations	Drake & Gahagan (2015)^17^	Working ‘Upstream’: Why we shouldn't use heterosexual women as health promotion change agents in HIV-prevention interventions aimed at heterosexual men.
		Gilbert (2012)^30^	‘Upstream/downstream’–locating the ‘social’ in health promotion and HIV/AIDS in South Africa?
‘Upstream’ ways of working	Political literacy and advocacy	Falk-Rafael & Betker (2012)^31^	Witnessing social injustice downstream and advocating for health equity upstream: ‘The trombone slide’ of nursing.
		Hayman et al. (2020)^32^	What knowledge is needed? Teaching undergraduate medical students to ‘go upstream’ and advocate on social determinants of health.
		McKinlay & Marceau (2000)^33^	To boldly go.
		Wallack & Thornburg (2016)^34^	Developmental origins, epigenetics and equity: moving upstream.
		Willen et al. (2017)^35^	Syndemic vulnerability and the right to health.
	Place-based, participatory and transformative action	Amaro (2014)^36^	The action is upstream: place-based approaches for achieving population health and health equity.
		Freudenberg et al. (2015)^19^	New approaches for moving upstream: How state and local health departments can transform practice to reduce health inequalities.
		Storey-Kuyl et al. (2015)^37^	Focusing ‘upstream’ to address maternal and child health inequities: two local health Departments in Washington State make the transition.
	Approaches underpinned by systems thinking and complexity science	Butterfield (2017)^16^	Thinking Upstream: A 25-year retrospective and conceptual model aimed at reducing health inequities.
		Carey and Crammond (2015)^38^	Systems change for the social determinants of health.
	Methodological pluralism	Asthana & Halliday (2006)^39^	Developing an evidence base for policies and interventions to address health inequalities: the analysis of ‘public health regimes’.
		Bambra et al. (2010)^40^	Reducing health inequalities in priority public health conditions: using rapid review to develop proposals for evidence-based policy.
		Pearce (1996)^41^	Traditional epidemiology, modern epidemiology, and public health.
		Smith et al. (2015)^42^	What kinds of policies to reduce health inequalities in the UK do researchers support?

## Results

**Fig. 1:**
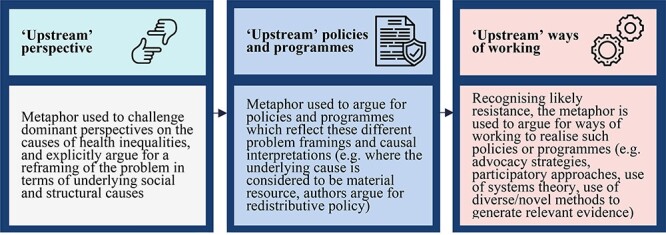
What is argued for through use of the ‘upstream–downstream’ metaphor?

The ‘upstream–downstream’ metaphor was used, firstly, to put forward what Entman^9^ would describe as a particular problem definition and causal interpretation of health inequalities, which centred the role of underlying social and structural causes, and which subsequently led authors to argue for a range of policies and programmes to address these causes. Recognizing the likely implementation challenges, a number of authors used the metaphor to provide detailed accounts of the process work needed to bring such changes to fruition. These three points will each be discussed in turn, illustrated using quotes and examples from the relevant articles. Figure 1 presents a summary of the findings.

### An ‘upstream’ perspective

Reflective of its dichotomous nature, the ‘upstream–downstream’ metaphor was used by all authors to firstly challenge and critique the dominant ‘downstream’ framing of the problem, and promote an alternative ‘upstream’ perspective. Baelum,^23^ for example, describes how ‘downstream’ interventions targeting inequalities in oral health have typically been underpinned by psychological theories that seek to explain behaviour in terms of individual beliefs and self-efficacy, without accounting for the role of wider social factors in shaping exposure to unhealthy environments and consequently health behaviour. In a similar way, SmithBattle^28^ describes how a ‘downstream’ approach to understanding social and health inequities associated with early childbearing works ‘to disregard the social context in which behaviour and choices are situated’, and treats people as though they exist ‘in a vacuum, disconnected from local settings and the larger socio-economic structures that organize and constrain individual actions’. Further emphasizing the importance of social relations, Drake and Gahagan^17^ detail how cognitive-behavioural interventions, designed to empower women to change their behaviour and reduce inequalities associated with HIV transmission, fail to appreciate that many women do not have a choice about their sexual and reproductive health as a result of gendered norms and power relations. As such, authors use the metaphor to promote an ‘upstream’ causal understanding or perspective on health inequalities, which underpins subsequent arguments for ‘upstream’ policies, programmes and ways of working.

### ‘Upstream’ policies and programmes

While critiques of the dominant ‘downstream’ framing were the same across articles, the ‘upstream’ policies and programmes discussed were found to reflect authors’ different perspectives on the nature of the problem. Some authors emphasized inequitable exposure to unhealthy environments, along with the disproportionate uptake and benefit of ‘downstream’ interventions amongst high income groups,^23–26^ as driving inequalities in lifestyle behaviours. ‘Upstream’ policies were consequently those which adopted a population approach and could ‘circumvent voluntary behaviour change’^25^ through regulating for the creation of healthier environments (e.g. smoke-free legislation). In contrast, where authors centred the importance of economic inequality in driving health inequalities,^18^^,^^27–29^ ‘upstream’ policies were those which involved redistribution and ‘fundamental social reform’^29^ to raise the incomes of low-wage workers, improve education and employment opportunities, and provide a safety net against poverty.^28^ Lastly, for those authors who centred the role of social norms and power relations in shaping personal autonomy and risk exposure,^17^^,^^30^ ‘upstream’ programmes were those which could be explicitly linked to a broader political project of, for example, achieving gender equity through wider social change. The example of microfinance initiatives was provided to illustrate how such programmes may work at the intersection of social and economic inequalities.^30^ While these authors focused on the rationale for specific policies/programmes, others used the metaphor to argue for ways of working needed for such actions to be realized.

### ‘Upstream’ ways of working

These authors, recognizing the inherent difficulties for most actors in ‘finding their way upstream’^19^ or in identifying ‘mechanisms for upstream change’,^16^ used the metaphor to detail the process work in which they could engage to bring about desired changes. For some, this took the form of arguing for the importance of political literacy and advocacy skills amongst both academic and professional workforces.^31–35^ Action of this kind was often not considered new, but rather was seen as a return to ‘traditional, premedicalized public health’, centred on ‘social and political activism’,^31^ and inspired by the actions of early leaders such as Florence Nightingale^31^ and John Snow.^33^ Borrowing a quote for Ilona Kickbusch, Willen et al.^35^ capture the principal concern amongst authors; that despite expanding knowledge, evidence and the best of intentions, real change will only come about when key actors have ‘a much better understanding of how politics works and what politics can achieve’.

For authors who situated their arguments within ideas of place-based, participatory and transformative action,^19^^,^^36^^,^^37^ the emphasis was on the nature of relationships between professional actors (e.g. local government departments) and communities. Freudenberg et al.^19^ for example, in describing action around living wages, mortgage foreclosures and air pollution, outlined the importance of a role reversal in successful campaigns where it was community organizers and grass roots coalitions who took the lead, with health departments and professionals acting in a supportive capacity (e.g. through furnishing relevant data and evidence). In two articles, authors drew on insights from systems thinking and complexity science in describing how change actually happens within institutions. Carey & Crammond,^38^ for example, reflecting on the mismatch between the ‘upstream–downstream’ dichotomy and systems frameworks, argue that it is counter-productive to think about ‘upstream’ change in terms of system levels, and what is needed is a better understanding of how different actions work to provoke change within systems. These insights are central to the Butterfield Upstream Model for Population Health,^16^ which explicitly seeks to guide nurses in ‘recalibrating systems’ for health equity through exploiting levers such as the beliefs and goals which sit at the core of their institutions.

Lastly were authors who used the metaphor to highlight how the prevailing model of knowledge and evidence production has given rise to an ‘inverse evidence law’,^43^ whereby the least evidence and research exists for policies and interventions thought to be most effective. These authors describe how ‘downstream’ causal perspectives, and the resulting interventions, are a more natural fit with this prevailing model in that they are easily defined, can be assessed using ‘hard’ outcome data, and are amenable to evaluation through controlled designs. Authors are consequently arguing for greater methodological pluralism to facilitate evidence production for ‘upstream’ policies, programmes, and ways of working, suggesting that the ‘emphasis should be on using appropriate methodology, rather than making the problem fit the method’.^41^

## Discussion

### Main finding of this study

This article illustrates how the ‘upstream–downstream’ metaphor is used initially as a reframing device, to critique and challenge the dominant ‘downstream’ framing of health inequalities, and promote an alternative problem definition and causal interpretation. This essential first step, of adopting an ‘upstream’ perspective or lens on the problem consequently opens up space to argue for a range of policies, programmes and ways of working. While the metaphor is a simple, and intuitively appealing one, the findings illustrate the breadth and complexity of disciplinary knowledge and ideas incapsulated in its use, along with the politically laden nature of many of the proposed actions. Additionally, it has shown how authors who, recognizing the likely difficulty for most actors in seeing how they might contribute to this agenda, are increasingly using the metaphor to go beyond describing the ‘what’, to detailing the ‘how’ of working ‘upstream’.

### What is already known on this topic

Despite extensive use of the ‘upstream–downstream’ metaphor, there has been limited critical reflection on its value, utility and function. One notable exception from Krieger^44^ raises concerns that the metaphor may promote a flawed and polarized understanding of causation, which artificially separates ‘downstream’ behavioural or biological causes from ‘upstream’ social factors, consequently obscuring their complex interaction across all levels. The metaphor has similarly been critiqued for its hierarchical conception of cause,^45^ which implies a passive and unidirectional flow through often concentric layers from ‘upstream’ to ‘downstream’, and is thus said to be unable to account for the complexity of social systems, in particular the role of feedback loops and individual agency.^46^ Citing McKinlay’s original article,^15^ Lundberg^47^ further suggests that using the metaphor to differentiate between determinants at micro- and macro-levels is misleading, as the upstream story is said to have originally been a metaphor for prevention in terms of the timing of intervention (i.e. before people fall into the stream), rather than the nature of intervention. Despite such reservations, however, the metaphor continues to be used and, as illustrated in this analysis, is no longer limited to simply differentiating between types or levels of determinants. Rather it is increasingly used in an argumentative way to promote alternative ways of thinking about health inequalities, and to provide strategic insights about the nature of action needed to realize change. The more pressing concern then perhaps is not whether the metaphor is an accurate depiction of causal pathways (as invariably all metaphors break down under scrutiny), but rather whether it ‘works’ amongst wider audiences to promote perspectives commensurate with established knowledge and evidence, and which ultimately engages people in seeing how they might contribute to this agenda in a meaningful way.

### What this study adds

The seemingly intractable nature of health inequalities has prompted authors to reflect on core disciplinary language and narratives. Concerns have been raised, for example, about the appropriateness of speaking of the social ‘determinants’ of health. Some authors have argued that this language can be easily reinterpreted within a ‘risk factor’ frame, thus promoting a reductionist approach to the problem of health inequalities.^48^ Others, however, have questioned the utility of employing the language of determinacy at all, as in its efforts to avoid victim blaming, it is said to deny individual agency and effectively reduce people to ‘puppets on strings’.^47^ Reservations have also been expressed about how well this language resonates with public audiences. Indeed, work underway by the Health Foundation and Frameworks Institute explicitly seeks to reframe the conversation around the social determinants of health in an effort to fill the ‘cognitive holes’ said to exist between expert and public understandings.^49^ The findings of this review make a valuable addition to such contemporary thinking and investigations by explicitly illustrating what some academic actors seek to achieve through use of this key public health concept.

### Limitations of this study

The arguments put forward in this paper are based on a sample of articles in which authors explicitly used the ‘upstream’ metaphor in arguing for action to reduce health inequalities, and the findings therefore cannot be said to be reflective of more general usage. It is impossible to do justice here to the depth of thinking and ideas presented in the original articles. The purpose of this paper however was not to produce an exhaustive account of the range of things that may count as ‘upstream’, but rather to illustrate what function the metaphor serves when employed by authors writing about health inequalities.

## Conclusion

At a time when oppositional forces are become increasingly adept at shaping debate, it is more important than ever to understand the extent to which favoured tools of communication ‘work’ (or indeed fail to work) to influence thinking and action. While not without its critics, the ‘upstream–downstream’ metaphor continues to be used extensively in the academic literature. However, the extent to which the metaphor resonates with wider audiences to evoke sentiments reflected in this account has yet to be established.

## Data availability

The data underlying this article will be shared on reasonable request to the corresponding author.

## Funding

This report is independent research funded by the National Institute for Health Research Applied Research Collaboration North West Coast (NIHR ARC NWC). NMcM is currently funded by an NIHR School for Public Health Research Postdoctoral Launching Fellowship. The views expressed are those of the author and not necessarily those of the National Institute for Health Research or the Department of Health and Social Care.

## Notes

**Naoimh E. McMahon**, , Postdoctoral Launching Fellow
